# DEK promoted EMT and angiogenesis through regulating PI3K/AKT/mTOR pathway in triple-negative breast cancer

**DOI:** 10.18632/oncotarget.21864

**Published:** 2017-10-17

**Authors:** Yang Yang, Meihua Gao, Zhenhua Lin, Liyan Chen, Yu Jin, Guang Zhu, Yixuan Wang, Tiefeng Jin

**Affiliations:** ^1^ Department of Pathology, Cancer Research Center, Yanbian University Medical College, Yanji 133002, China; ^2^ Department of Internal Medicine, Yanbian University Hospital, Yanji 133000, China; ^3^ Department of Biochemistry and Molecular Biology, Yanbian University Medical College, Yanji 133002, China; ^4^ Department of Anatomy, Histology and Embryology, Yanbian University Medical College, Yanji 133002, China

**Keywords:** DEK, prognosis, angiogenesis, metastasis, triple-negative breast cancer

## Abstract

Triple-negative breast cancer (TNBC) is a highly aggressive subtype of breast cancer associated with poor prognosis. As an oncogene, DEK involves in regulation of various cellular metabolisms and plays an important role in tumor growth and progression. Increasing evidences suggested that abnormal expression of DEK is closely related to multiple malignant tumors. However, the possible involvement of DEK in epithelial to mesenchymal transition (EMT) and angiogenesis in TNBC remains unclear. In the present study, we revealed that the over-expression of DEK was significantly correlated with clinical stage, differentiation, and lymph node (LN) metastasis of TNBC and indicated poor overall survival of TNBC patients. Moreover, we demonstrated that DEK depletion could significantly reduce cell proliferation, migration, invasion and angiogenesis *in vitro*. We also found that DEK promoted cancer cell angiogenesis and metastasis by activating the PI3K/AKT/mTOR pathway. Furthermore, we revealed the inhibitory effect of DEK depletion on tumor growth and progression in a xenograft tumor model in mice. These data indicated that DEK promotes TNBC cell proliferation, angiogenesis, and metastasis via PI3K/AKT/mTOR signaling pathway, and therefore, it might be a potential target in TNBC therapy.

## INTRODUCTION

TNBC accounts for ~15% of invasive breast cancers, which is often highly proliferative, poorly differentiated and associated with poor prognosis [1, 2]. Although some patients respond well to cytotoxic chemotherapies, relapses are frequent and resistance to available treatments occurs almost without exception in the metastatic setting [3]. Because of the lack of targeting agents and limited therapeutic options, treatment of TNBC remains a great clinical challenge. To date, increasing evidences suggested that aberrant activation and dysfunction of crucial genes result in progression of TNBC [4, 5]. Therefore, it is critical to identify specific molecular markers which could serve as clinical/prognostic factors and elucidate the molecular mechanisms involved in TNBC.

The oncogene DEK, a CAN nucleoporin fusion protein, which was found in a subtype of acute myeloid leukemias, was identified as a non-histone chromosomal factor [6]. As a versatile nuclear protein, DEK can bind chromatin and involve in various fundamental nuclear processes, including DNA damage repair [7], DNA replication [8], mRNA splicing [9], transcriptional regulation [10], differentiation [11] and apoptosis [12]. Additionally, it is reported that up-regulation of DEK expression has been implicated in many types of tumors [13, 14]. Nakamura *et al*. first mentioned that DEK was up-regulated in pancreatic ductal adenocarcinoma (PDAC), and demonstrated as a metastasis associated gene in PDAC [15]. Subsequently, Adams *et al*. reported that DEK was found to be over-expressed in head and neck squamous cell carcinoma (HNSCC), and its expression was closely related to tumor apoptosis and poor prognosis [16]. These results indicated that targeting DEK might be an effective anti-tumor therapy strategy.

Accumulating evidences had shown that tumor angiogenesis and EMT are key targets for modern research in antitumor therapy. Recent study had shown that DEK as a novel coactivator for HIF-1a in regulation of VEGF transcription and a promoter of angiogenesis in breast cancer [17]. Yu *et al*. also reported that DEK might promote hepatocellular carcinoma cell (HCC) migration and EMT through the regulation of β-catenin/E-cadherin signaling [18]. These results indicated that DEK participated in cancer cell angiogenesis and EMT. However, the exact role and mechanism of DEK in TNBC remains largely unknown.

In this study, we demonstrated that DEK knockdown attenuated cell proliferation, migration, invasion, and angiogenesis *in vitro*, and delayed tumor growth and mouse caudal vein metastasis in xenograft mouse model. In addition, we found that silencing of DEK suppressed TNBC cell tumorigenesis and EMT through the regulation of PI3K/AKT/mTOR signaling pathway. These findings may provide the potential use of DEK as a therapeutic target of TNBC.

## RESULTS

### DEK expression is significantly up-regulated in TNBC

**Figure 1:**
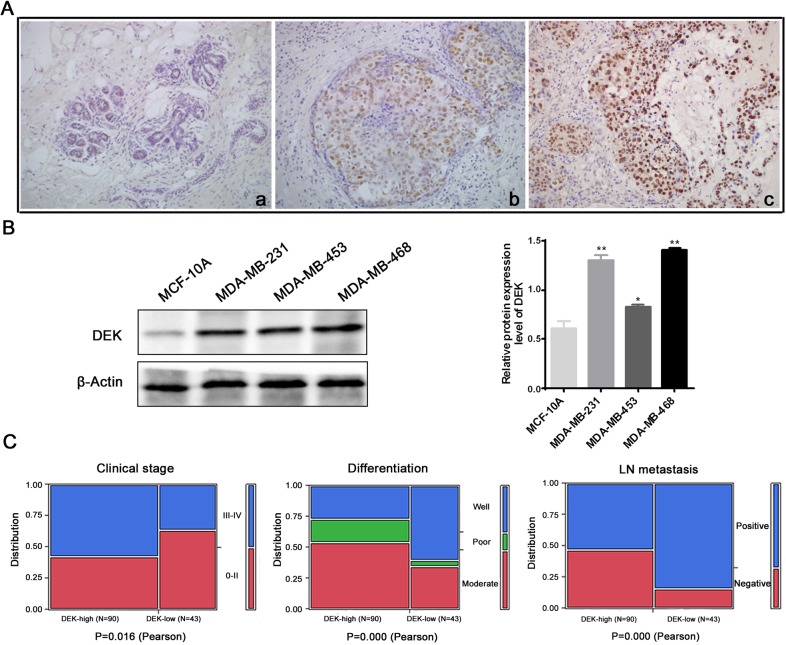
DEK expression is significantly up-regulated in TNBC **(A)** DEK immunohistochemical staining in representative cases from each breast cancer lesion (original magnification, 200×). **(B)** Western blot assays performed on TNBC cell lines as indicated. **(C)** The relationships between DEK expression and the clinicopathologically significant aspects of TNBC.

To determine the biological role of DEK in human TNBC progression, DEK expression was first examined in 133 pairs of TNBC and 55 normal breast tissues by IHC. The IHC staining showed that DEK is mainly located to the nucleus of TNBC cells (Figure 1A). Here, the positive rate of DEK expression in TNBC was 86.5% (115/133), which was significantly higher than that in ductal carcinoma in situ (DCIS) tissues (positive rate: 66.0%) and adjacent non-tumor tissues (positive rate: 23.6%) (*P*<0.001, respectively). Similarly, the strong positive rate of DEK expression in TNBC (67.7%, 90/133) was also significantly higher than that in DCIS (36.2%, 17/47) and adjacent non-tumor tissues (12.7%, 7/55) (*P*<0.001, *P*<0.001, respectively) (Table 1). Then, western blot was performed to determine the expression level of DEK in TNBC cell lines. Protein levels of DEK in TNBC cell lines (MDA-MB-468, MDA-MB-231, and MDA-MB-453) were noticeably higher than it in normal human breast epithelial cells MCF-10A (Figure 1B). Furthermore, Pearson's χ2 test showed that the abnormal expression of DEK protein was significantly associated with clinical stage (*P*=0.016), differentiation (*P*=0.000) and lymph node metastasis (*P*=0.000), but not with other clinicopathological parameters, including age, menopausal status or tumor size (Table 2, Figure 1C).

**Table 1:** DEK protein expression in TNBC

Diagnosis	No. of cases	DEK protein expression				Positive rate	Strongly positive rate
		-	+	++	+++		
Adjacent non-tumor	55	42	6	7	0	23.64%	12.7%
DCIS	47	16	14	11	6	66.0%^**^	36.2%^**^
TNBC	133	18	25	38	52	86.5%^**^	67.7%^**^

^**^P<0.01: compared with adjacent non-tumor.

DCIS: ductal carcinoma in situ.

TNBC: triple-negative breast cancer in situ.

**Table 2:** Relationship between DEK protein overexpression and the clinicopathological features of TNBC

Clinical features	No. of cases	Strongly positive cases (%)	χ^2^	P value
Age			0.258	0.611
≥50	70	46(65.7%)		
<50	63	44 (69.8%)		
Menopausal status			0.474	0.491
Premenopausal	81	53(65.4%)		
Postmenopausal	52	37 (71.2%)		
Tumor size			0.063	0.087
T1	71	50 (70.4%)		
T2	62	40 (64.5%)		
Tumor differentiation			15.284	0.000^**^
Well	50	24(48.0%)		
Moderate	64	49(76.6%)		
Poor	19	17(89.5%)		
Clinical stage			8.836	0.003^**^
0-II	68	38 (55.9%)		
III-IV	65	52 (80.0%)		
LN metastasis			8.020	0.005^**^
Postive	73	57 (78.1%)		
Negtive	60	33 (55.0%)		

^*^p<0.05 and ^**^ *p*<0.01.

Previous studies demonstrated that MVD which is measured by CD34 was a reliable method to evaluate angiogenesis of breast cancer [19, 20]. Hence, to determine the relationship between DEK expression and microvessel density (MVD), we used anti-CD34 mAb immunostaining in TNBC tissue. The results showed that MVD in DEK positive expression TNBC was 43.21±12.431, which was significantly higher than that in DEK negative expression TNBC (18.89±5.711). These data indicated that MVD was significantly associated with DEK (r=0.583, *p*=0.000) (Supplementary Figure 1).

### Elevated DEK expression correlated with poor prognosis in TNBC patients

**Figure 2:**
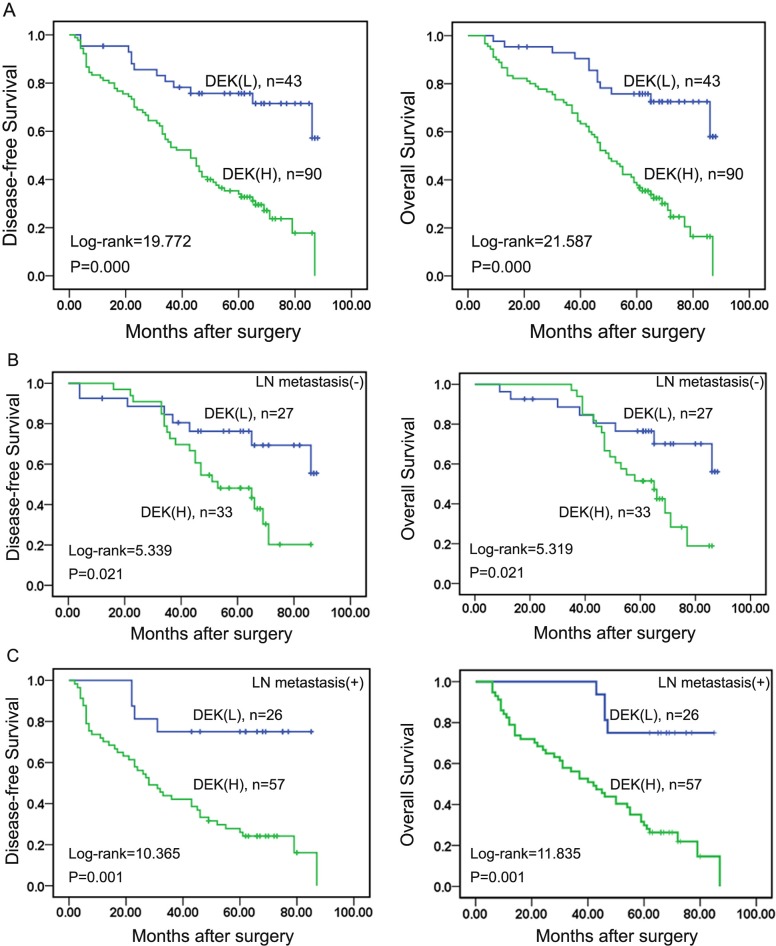
DEK over-expression predicted poor prognosis of TNBC **(A)** Kaplan-Meier survival curves in TNBC patients with high and low DEK expression. **(B)** Kaplan-Meier survival curves in the TNBC patients without lymph node metastasis. **(C)** Kaplan-Meier survival curves in the TNBC patients with lymph node metastasis.

To further elucidate the relationship between DEK expression and the prognosis of TNBC patients, we used Kaplan-Meier method to analyze the disease-free survival (DFS) and overall survival (OS) of 133 TNBC patients. Here, Kaplan-Meier survival analysis showed that patients with high DEK expression had significantly shorter survival time (both *P*<0.001) (Figure 2A). Equally, the patients with over-expression of DEK had shortened DFS and OS compared with low DEK expression in LN metastasis (−) and LN metastasis (+) TNBC patients (both *P*<0.05) (Figure 2B and 2C). Moreover, clinical stage, LN metastasis, and DEK expression status were correlated with shorter survival time in univariate analysis (Table 3). Multivariate Cox regression analysis further substantiated that DEK was an independent risk factor for lower DFS and shortened OS in patients with TNBC (Table 3).

**Table 3:** Univariate analysis of clinicopathological factors for the overall survival rate of 133 patients with TNBC

Characteristics	B	SE	Wald	HR	95%CI		P value
					Lower	Upper	
Univariate							
Age	0.114	0.175	0.423	1.120	0.795	1.578	0.515
Menopausal status	0.288	0.181	2.530	1.333	0.935	1.900	0.112
Tumor size	0.281	0.179	2.479	1.325	0.933	1.880	0.115
Tumor differentiation	0.171	0.124	1.906	1.186	0.931	1.511	0.167
Clinical stage	0.729	0.181	16.278	2.073	1.455	2.953	0.000^**^
LN metastasis	0.408	0.178	5.253	1.504	1.061	2.131	0.022^*^
DEK	0.629	0.191	10.812	1.875	1.289	2.728	0.001^**^
Multivariate							
LN metastasis	0.212	0.182	1.349	1.236	0.864	1.767	0.246
Clinical stage	0.705	0.187	14.2030	2.023	1.402	2.918	0.000^**^
DEK	0.596	0.201	8.798	1.815	1.224	2.690	0.003^**^

^*^Significant difference.

B: Coefficient; SE: standard error, Wald: Waldstatistic; HR: hazard ratio; CI: confidence interval.

### DEK promoted TNBC cell proliferation, migration and invasion *in vitro*

**Figure 3:**
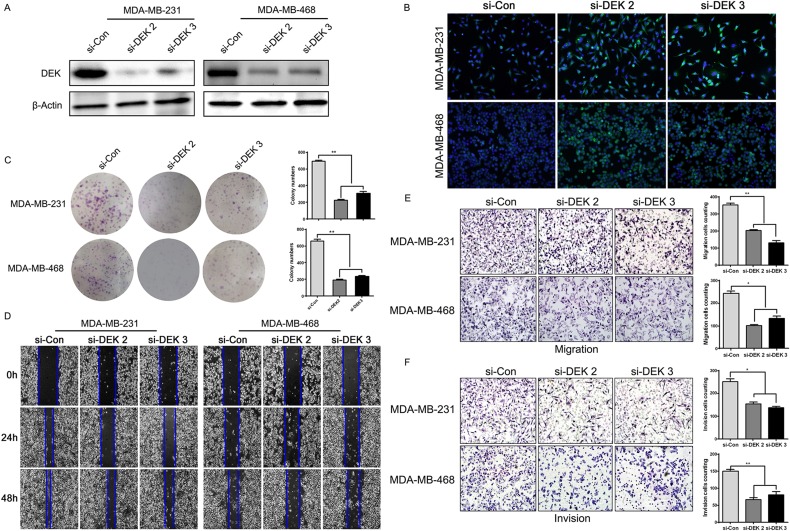
DEK knock-down attenuated the ability of proliferation, migration and invasion of TNBC cells *in vitro* **(A)** DEK protein expression was inhibited in si-DEK-transfected MDA-MB-231 and MDA-MB-468 cells compared with si-control cells, as demonstrated by western blot. **(B)** Proliferating capability of transfected cells was evaluated using CFSE incorporation. **(C)** The number of colonies per plate was counted. **(D)** Scratch wound-healing assay was used to determine the effects of si-DEK on TNBC cells motilities. **(E and F)** Migration of DEK knock-down cells was measured by transwell migration assay. The invasive abilities of TNBC cells were determined in Boyden chamber assay after transfection with si-DEK or si-control. The results were quantitated by counting invading cells in five randomly chosen high-power fields for each replicate. *P* values were obtained using Mann-Whitney U Test. (original magnification, 100×).

To investigate the impact of DEK in TNBC progression, DEK was knocked down in over-expressed TNBC cell lines (MDA-MB-468 and MDA-MB-231). The cells were transfected with non-targeting siRNA (si-control) or two different DEK-specific lentiviral siRNA, and the silencing effects were confirmed by western blot (Figure 3A). The results revealed that DEK knockdown led to a remarkable inhibition in cell proliferation as determined by CFSE and colony formation assay (Figure 3B and 3C). Moreover, we used wound-healing and transwell assays to investigate the role of DEK in TNBC cells motility and invasion. The results also showed that silencing of DEK reduced the migration and invasion ability of TNBC cells (Figure 3D-3F). These results demonstrated that DEK depletion increased cell-cell contacts and suppressed cell proliferation, oncogenic transformation, migration and invasion potentials, suggesting an important role of DEK in TNBC progression.

To further investigate the effects of DEK on MCF10A cells, we transfected the cell with DEK-overexpression plasmid. Western blot was used to confirm the increased levels of DEK in cells transfected with DEK vector (Supplementary Figure 2A). MCF10A cells are typically epithelial-like. Interestingly, when DEK was overexpressed, these cells rendered a mesenchymal morphology (Supplementary Figure 2B) and acquired migratory capability (Supplementary Figure 2C). In addition, compared with control vector, the western blot assay showed that DEK overexpression up-regulated the expression of mesenchymal marker proteins: Vimentin, Snail, and Slug while down-regulated epithelial marker, E-cadherin (Supplementary Figure 2D). These results suggest that DEK overexpression promotes tumorigenic phenotypes in MCF10A cells.

### DEK promoted TNBC cell migration and invasion via activating EMT

**Figure 4:**
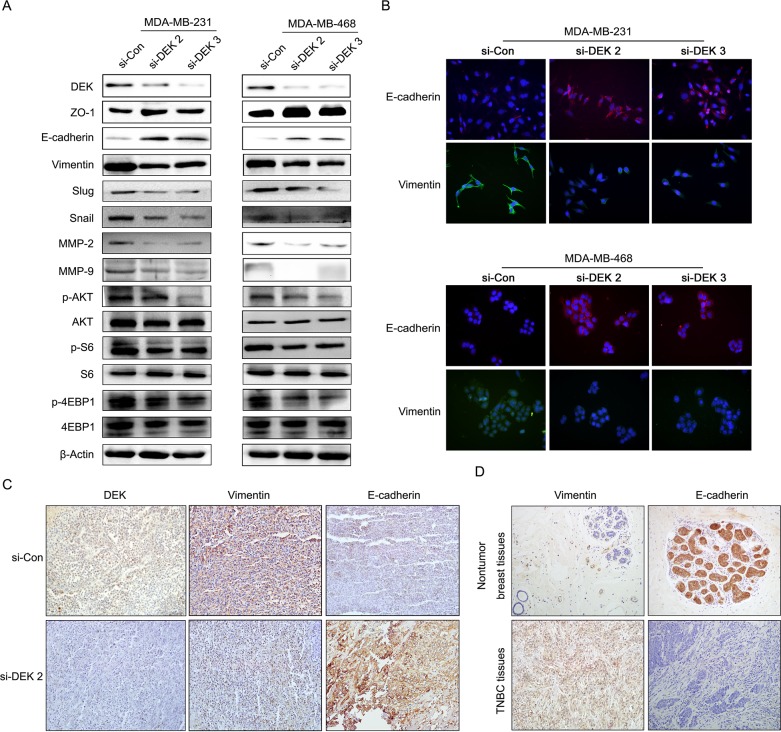
DEK regulated TNBC cell migration and invasion via activating EMT **(A)** Relative expression levels of DEK, AKT, phosphorylated AKT, S6, phosphorylated S6, 4EBP1, phosphorylated 4EBP1, EMT markers in TNBC cells with si-control or si-DEK, respectively. **(B)** Expression of EMT markers was detected by IF staining in TNBC cells transfect with si-control and si-DEK (original magnification, 100×). **(C)** Immunohistochemical staining for E-cadherin and Vimentin protein in tumor specimens from xenografts (original magnification, 100×). **(D)** Proteins expression of E-cadherin and Vimentin in TNBC tissues and adjacent normal tissues (original magnification, 200×).

Previous studies showed that cell migration and invasion are associated with altered levels of EMT biomarkers [21]. To determine whether DEK participated in EMT process of TNBC, we used western blot to detect the expression of EMT markers. Our results showed that silencing of DEK up-regulated epithelial marker (E-cadherin and ZO-1), while down-regulated mesenchymal marker (Vimentin, slug, snail) and MMPs (MMP-2, and MMP-9) (Figure 4A). Using IF staining, we also analysed the expression levels of EMT markers. Consistent with the results of western blot, silencing of DEK significantly increased E-cadherin expression and decreased Vimentin in TNBC cells (Figure 4B), suggesting that silencing of DEK inhibited EMT process in TNBC cells *in vitro*.

To confirm these findings *in vivo*, IHC was performed in the transplanted tumor tissues of TNBC cells. Consistently, we found that silencing of DEK decreased Vimentin expression, while increased E-cadherin expression (Figure 4C). IHC stainings further showed that E-cadherin was decreased, but Vimentin was increased in the transplanted tumor tissues of TNBC cells (Figure 4D), which consisted with the *in vitro* results. Overall, DEK inhibition reversed the process of EMT, suggesting that DEK could be a novel promoter of the EMT in TNBC cells.

### Depletion of DEK suppressed TNBC cell angiogenesis

**Figure 5:**
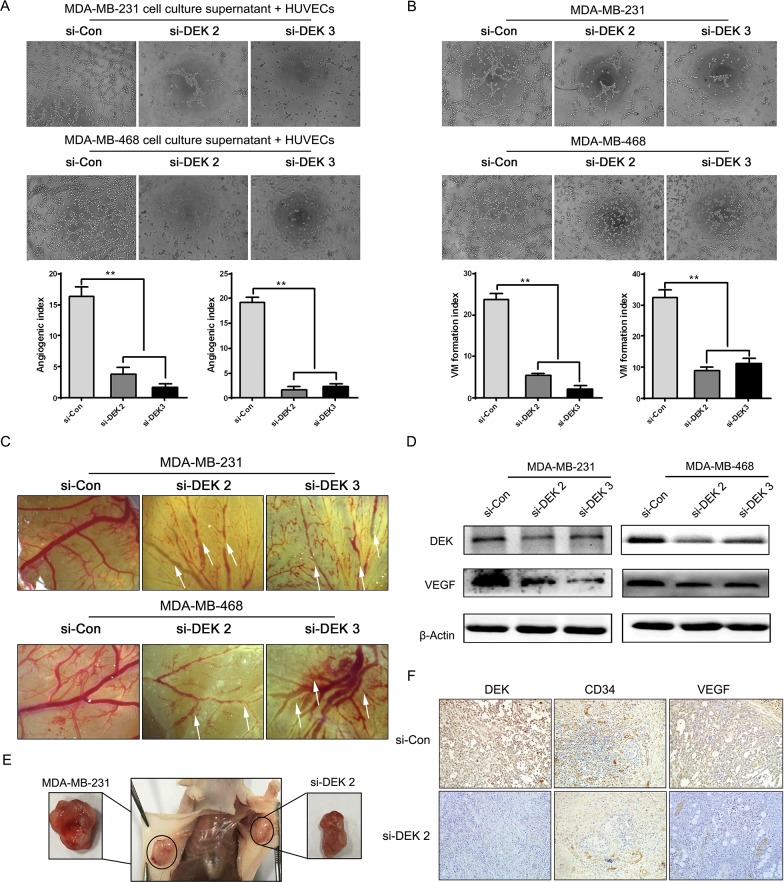
Silencing of DEK suppressd angiogenesis *in vitro* and *in vivo* **(A)** Tube formation assay was performed with HUVECs cells incubated in culture supernatant fluid of TNBC cells 48 h after transfection with the si-control and si-DEK (original magnification, ×100). **(B)** Vasculogenic mimicry formation assay was performed with TNBC cells (original magnification, ×100). **(C)** The new blood vessel formation in CAM was quantified. **(D)** Relative expression levels of DEK and VEGF in TNBC cells with si-control or si-DEK, respectively. **(E)** Images of blood vessels between si-control and si-DEK tumors and surrounding tissue. **(F)** Immunohistochemical staining for CD34 and VEGF protein in tumor specimens from xenografts (original magnification, 100×).

DEK have been reported to activate VEGF expression, which play a key role in cancer neoangiogenesis [17]. To understand the function of DEK in TNBC angiogenesis, we further determined the effects of DEK on capillary tube formation of HUVECs *in vitro*. We found that DEK depletion in TNBC cells decreased microtubule formation ability of HUVECs *in vitro* (Figure 5A). Vasculogenic mimicry analysis was also performed, and we found that silencing of DEK reduced 62% mimicry formation of TNBC cells (Figure 5B). Additionally, we utilized the chick embryo chorioallantoic membrane (CAM) assay to further characterize the silencing effect of DEK on TNBC angiogenesis, and found that the DEK-siRNA groups had significant damaged blood vessels formation when compared with the control groups (Figure 5C). Additionally, we found that silencing of DEK down-regulated the marker of angiogenesis: VEGF (Figure 5D). Subsequently, we detected the growth condition of blood vessels after the experimental animals were sacrificed. Silencing of DEK suppressed angiogenesis, resulting in a decrease in the number and size of blood vessels in the tumors and surrounding tissues (Figure 5E). As expected, we also found significantly decreased VEGF expression in xenografts of MDA-MB-231 cells with DEK knockdown by immunostaining (Figure 5F). In addition, the amount of microvessel density (MVD) determined using anti-CD34 mAb immunostaining in the the transplanted tumor tissues of TNBC cells (Supplementary Figure 4). MVD counting was shown that MVD in xenograft tumor tissues was 22.14±2.795, which was significantly higher than that in si-control group (4.57±1.718). MVD was significantly associated with anti-DEK (r=0.971, *p*=0.000) (Supplementary Figure 4). Together, our data indicated that depletion of DEK suppressed TNBC angiogenesis.

### DEK mediated epithelial mesenchymal transition and angiogenesis in TNBC cells occurs via the PI3K/AKT/mTOR signaling pathways

Previous articles reported that PI3K/AKT/mTOR signalling pathway played a crucial role in EMT process and angiogenesis [22, 23]. Thus, to elucidate whether PI3K/AKT/mTOR pathway involved in DEK-induced tumor growth and progression in TNBC cells, we detected the expression level of PI3K/AKT/mTOR signaling pathway and downstream molecules by western blot. The results showed that silencing of DEK in TNBC cells caused a significant decrease in AKT, S6, 4EBP1 phosphorylation levels (Figure 4A). These results suggested that silencing of DEK suppresses EMT, at least in part through the regulation of PI3K/AKT/mTOR signaling pathway.

To evaluate whether the effects of DEK on cell migration and angiogenesis were dependent on the PI3K/AKT/mTOR pathway, MDA-MB-468 and MDA-MB-231 cells with stably over-expressed DEK (Supplementary Figure 3A), were treated with LY290042 (PI3K inhibitor) or Rapamycin (mTOR inhibitor) for 48 h. Inhibition of AKT or mTOR exhibited significant lower capacities of migration (Supplementary Figure 3B). As illustrated in Supplementary Figure 3C, DEK overexpression significantly promoted HUVEC tube formation in MDA-MB-468 and MDA-MB-231 cells, compared with control vector. As expected, inhibition of AKT by LY290042 or of mTOR by Rapamycin reversed the effects on Vimentin, Slug and VEGF up-regulation and E-cadherin down-regulation induced by DEK (Supplementary Figure 3D). These results indicated that DEK induce cell migration, angiogenesis, and EMT through PI3K/AKT/mTOR signaling pathways.

### Silencing of DEK suppressed tumors growth, and metastasis in tumor xenografts

**Figure 6:**
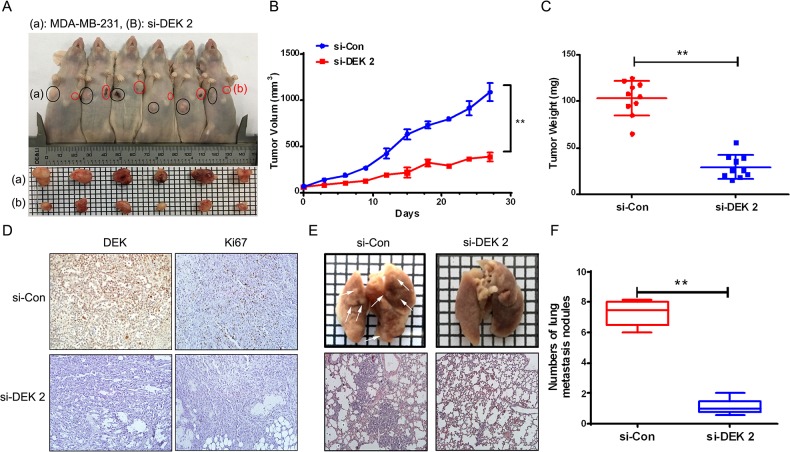
Silence of DEK inhibited tumor growth and metastasis in mouse xenograft model **(A)** Images of tumors formed by subcutaneous injection of si-control cells (a) or si-DEK cells (b) in Balb/c nude mice. **(B and C)** Si-DEK tumors exhibited statistically significant decrease in tumor growth and tumor weight when compared to si-control tumors (^**^P<0.01). **(D)** Expression of DEK and Ki67 in xenograft tumor tissues by IHC. **(E and F)** Representative gross and H&E-staining pictures (E) and the number of lung surface metastatic foci detected in each group (F) (^**^P<0.01). *P* values were obtained using Mann-Whitney U Test.

We further confirmed the effects of DEK on tumorigenesis and metastasis using xenograft mouse model of MDA-MB-231 cells transfected with si-control or si-DEK 2 to verify the physiological relevance of our *in vitro* observations. Here, we showed that the si-DEK 2 tumors exhibited a significant decrease in volume (*P*<0.05, *P*<0.01) and weight (*P*<0.01) as well as a marked decrease in expression of Ki-67 compared with the si-control group in Figure 6A-6D. We then investigated whether silencing of DEK suppressed lung metastases *in vivo*. As shown in Figure 6E and 6F, silencing of DEK had fewer lung metastases than the mice injected with the si-control cells. These results indicated that silencing of DEK in MDA-MB-231 cells suppressed tumor growth and lung metastasis *in vivo*.

## DISCUSSION

A growing body of evidence has identified that genes specifically up-regulated or downregulated in TNBC tissues can be considered as early diagnostic markers, prognostic markers, and therapeutic targets [24]. DEK is such a gene that is highly up-regulated in various types of tumors [13]. However, little is known about its expression in TNBC, with even less clarity on its underlying mechanisms. In our present study we first determined that DEK expression was markedly higher in TNBC tissues and cells, and correlated with clinical stage, differentiation, and lymph node metastasis. A multivariable Cox's regression analysis indicated that DEK was a significant risk factor of OS and DFS.

DEK is an oncoprotein involved in many aspects of cellular functions, for example, DNA repair, replication, and transcriptional control [8, 25]. Many researchers considered DEK as a candidate anti-cancer target. Yamazaki *et al*. reported that DEK depletion enhances apoptosis and chemosensitivity of canine transitional cell carcinoma cells [26]. Chen *et al*. also found that DEK inhibited colon cancer cells growth by blocking cell cycle and inducing cell apoptosis [27]. In addition, Privette *et al*. found that knockdown of DEK decreased invasion and mammosphere formation of breast cancer cell viability *in vitro* [28]. In accordance with these results, we found that depletion of DEK exhibited strong inhibitory effects on TNBC proliferation, invasion and migration *in vitro*. In addition, we also found that DEK knockdown inhibited cell proliferation and metastasis *in vivo*, suggesting the oncogenic role of DEK in TNBC, consistent with its role in other cancers [29].

EMT is a progressive biological phenomenon that epithelial cells gradually change into mesenchymal cell phenotype, resulting in enhanced invasion and metastasis. Recent studies indicated that EMT is relevant to the invasion and metastasis of TNBC cancer [30, 31]. Yu *et al*. DEK-dependent migration and EMT occurs via β-catenin/E-cadherin signalling in HCC cells [18]. In current study, we found that silencing of DEK inhibited EMT process by reducing expression of the mesenchymal markers (Vimentin, Slug, and Snail) by elevating expression of the epithelial marker (E-cadherin and ZO-1). Besides, DEK overexpression promotes tumorigenic phenotypes in immortalized human mammary epithelial MCF10A cells. These results suggested that DEK promoted TNBC cell migration and invasion via activating EMT. A recent study indicated that silencing of Scrb causes E-cadherin accumulation into perinuclear vesicles [32]. Elzagheid *et al*. found that E-cadherin proteolysis and nuclear translocation was associated with colorectal cancer progrsssion, and might be a new biomarker for diagnosis and prognosis of colorectal cancer [33]. Here, we demonstrated that the depletion of DEK increased membranous expression of E-cadherin in TNBC cells. Accordingly, the mechanism by which the E-cadherin molecule is translocated to the cytoplasm and nucleus in TNBC cells needs the further study to clarify. Consistently, *in vivo* experiments, increased expression of E-cadherin accompanied by decreased expression of Vimentin were observed in the mice bearing the MDA-MB-231 xenografts and TNBC tissues samples by IHC. Similarly, Ito *et al*. had been shown that downregulate the expression of E-cadherin can inhibit lung metastases in TNBC [34]. These results indicated that DEK promote invasion and migration via inducing EMT process in TNBC.

Angiogenesis plays a central role in the development and progression of malignant tumors since adequate blood supply is necessary for cancer cell growth, invasion and metastasis [35]. As a key regulator of angiogenesis, VEGF is important not only for proliferation and migration of endothelial cells but also for proliferation and/or survival of solid cancer cells in an autocrine and/or paracrine manner [36, 37]. Zhang *et al*. had shown that DEK directly binds to the DRE of the VEGF promoter, which enhances the cell proliferation, migration, tube formation of vascular endothelial cells and tumor angiogenesis as well [17]. CD34 is a glycosylated transmembrane cell surface glycoprotein which has been used to measure angiogenesis [38]. During the development of human vascular tumors, over-expression of CD34 could promote tumor angiogenesis [39]. In this study, we observed that silencing of DEK inhibited microtubule formation, and vasculogenic mimicry *in vitro* and decreased micro-vessel density *in vivo*. Moreover, we also found the important roles of DEK in inhibiting angiogenesis via VEGF and CD34 *in vitro* and *in vivo*. These findings suggested that depletion of DEK attenuated angiogenesis of TNBC.

The PI3K/AKT/mTOR signaling is a critical pathway in cell proliferation, survival, neovascularization and tumor growth [40, 41]. Previous studies have identified that PI3K/AKT/mTOR signaling plays an irreplaceable role in the progression and maintenance of breast cancer [42]. Deregulation of PI3K/AKT/mTOR signaling occurs in almost all cases of advanced TNBC [43]. We showed that phosphorylated levels of AKT, mTOR, S6 were enhanced by DEK overexpression and inhibited by DEK silencing, which is consistent with previous reports [44]. Furthermore, we investigated if PI3K/AKT/mTOR signaling is responsible for DEK-mediated acceleration of tumor cell migration, angiogenesis, and EMT. Blocking PI3K/AKT/mTOR pathway using special inhibitors significantly attenuated DEK-enhanced migration and angiogenesis of TNBC cells. More importantly, LY290042 or Rapamycin treatment inhibited the activation of VEGF, slug, and snail as well as inactivation of E-cadherin by DEK. Therefore, our data support that activation of PI3K/AKT/mTOR signalling pathways was required for DEK-stimulated cell migration, angiogenesis, and EMT of TNBC cells.

**Figure 7:**
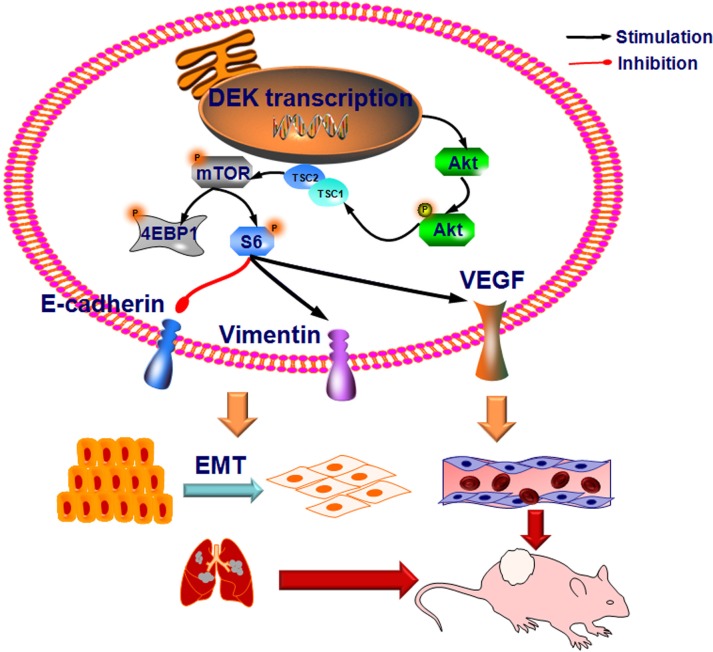
Proposed models on the carcinogen function of DEK in EMT, angiogenesis and metastasis of TNBC

In summary, our study demonstrated that DEK was overexpressed in TNBC and played a major role in the progression and metastasis of TNBC. Of note, we have also provided insight into the underlying mechanism, and obtained evidence that DEK regulates the EMT and PI3K/AKT/mTOR signaling pathways involved in metastasis and angiogenesis (Figure 7). Together, these observations indicate that DEK may serve as a therapeutic target for TNBC treatments.

## MATERIALS AND METHODS

### Ethics statement

This research complied with the Helsinki Declaration and was approved by the Human Ethics Committee and the Research Ethics Committee of Yanbian University Medical College. Patients were informed that the resected specimens were stored by the hospital and potentially used for scientific research, and their privacy could be maintained. Follow-up survival data were collected retrospectively through medical record analysis.

### Clinical samples

The study of 133 paraffin embedded TNBC samples, 47 DCIS samples, 55 adjacent non-tumor tissues were conducted. These samples were selected randomly from patients who underwent surgery between 2002 and 2009, with strict follow-up for survival status. Clinicopathological classification and staging were determined according to the American Joint Committee on Cancer (AJCC) criteria.

### Cell culture

The human TNBC cell line MDA-MB-231, MDA-MB-468, MDA-MB-453 and normal non-malignant breast cells (MCF-10A) (American Type Culture Collection, Manassas, VA, USA) was cultured-in Dulbecco's Modified Eagle Medium (DMEM) containing 10% fetal bovine serum (FBS) and streptomycin-penicillin (100 U/mL). Cells were incubated at 37°C in an atmosphere of 5% CO_2_.

### Antibodies

Antibodies against DEK, ZO-1, E-cadherin, Vimentin, Snail, Slug, p-S6, S6, p-Akt, Akt, p-4EBP-1, 4EBP-1 and β-Actin were purchased from Cell Signaling Technology (Boston, USA). Antibody against MMP-2 was purchased from BOSTER (Wuhan, China). MMP-9 and Slug were purchased from Affinity (Cincinnati, USA). Antibody against VEGF, CD34 was purchased from Abcam (Cambridge, UK).

### Transfection

We purchased three different DEK siRNA, including si-RNA1, si-RNA2, and si-RNA3, from RIBOBIO (China). According to the KD effect, si-RNA2 and si-RNA3 was used in this study. The sequence of si-RNA2 (si-DEK 2) was 5′-CGAACCAAAUGUCCUGAAA-3′. The sequence of si-RNA3 (si-DEK 3) was 5′-UGUCCUCAUUAAAGAAGAA-3′. Additionally, control siRNA (si-control) was also used in this study. Cells were transfected with 30 nM siRNA using Lipofectamine 3000 (Invitrogen) according to the manufacturer's instructions.

### Wound healing assay

Cells were plated into six-well plates at 95% confluence in complete tissue culture medium. After the cells became confluent, cell wounds were created by scratching cells using a micropipette tip. The medium was then immediately replaced, and spontaneous cell migration was monitored using a Nikon inverted microscope at 0 h, 24 h and 48 h. The distance of wound closure was measured in three independent wound sites per group. The wound gaps were measured at each time.

### Immunofluorescence

Cells grown in six-well culture slides fixed with 4% paraformaldehyde for 15 min, permeabilized with 0.5% Triton X-100 (CWBIO, China) and blocked with 3% BSA for 2 h. Cells were incubated with primary antibody in 3% BSA at 4°C overnight, washed three times with PBS, incubated with Alexa Fluor 488 or Alexa Fluor 546-labeled secondary antibody (Invitrogen) in 3% BSA for 2 h, and then analyzed by Leica SP5II confocal microscope.

### Migration assay

The migration assay used 24-well Millicell (MILLIPORE) with 8-μm PET. Cells were seeded into the upper insert in serum-free media, while media containing 10% FBS was added to the lower chambers as a chemoattractant for 12 h. The cells were removed from the upper surface of the filter by scraping with a cotton swab. Cells that infiltrated through the filter were fixed and stained with Giemsa. The images were taken with OLYMPUS BX53.

### Invasion assay

The invasion assays used 24-well BD BioCoat Matrigel invasion chambers with 8-μm pore inserts (BD). Cells (5×10^4^) suspended in serum-free media were seeded in the upper inserts, while media containing 10% FBS was added to the lower chambers as a chemoattractant. The cells were removed from the upper surface of the filter by scraping with a cotton swab after 48 h in culture. Cells that infiltrated through the filter were fixed and stained with Giemsa. The mean values of the results obtained with the three chambers were used in the analysis.

### Colony-forming assay

Single-cell suspension (1000 cells per well) were seeded in six-well plates and incubated for 2 weeks. The cells were fixed by ice-cold methanol and stained by Giemsa for 25 mins. Washing 30 mins with tap water, and the colonies (more than 50 cells) were counted directly on the plate. Statistical significant was calculated from each three independent experiments.

### Carboxyfluorescein diacetate succinimidyl ester (CFSE) and cell proliferation

Cell proliferation is evaluated by the CellTrace™ CFSE cell proliferation kit according to manufacturer protocol. Briefly, mammospheres were dissociated and incubated with PBS containing 5 μM CFSE/1×10^6^ cells and 0.1% BSA, for 15 mins, at 37°C. The reaction was stopped with cold culture medium and after washing with the medium, cells were seeded at 10000 cells/cm^2^ and treated in culture. After 24 h, mammospheres were allowed to adhere on poly-L-lysine coated glass coverslips and fixed in 4% paraformaldehyde in PBS, for 10 minutes, at RT. Cell nuclei were stained with DAPI (0.5 μg/mL). Coverslips were mounted with vectashield mounting medium and examined at a Leica TCS SP5 confocal microscope (Mannheim, Germany).

### Western blot

Immuno-blot analysis was performed as previously described [45]. Briefly, cells were harvested and lysed with RIPA buffer containing 1 mM PMSF and a protease inhibitor cocktail (Roche, Germany). The protein concentration of each sample was measured using BCA protein assay kit (Pierce, Rockford, Illinois). Protein samples (20 μg/lane) were electrophoresed (Bio-Rad, Hercules, CA) on 8%-12% SDS polyacrylamide gel and transferred to PVDF membranes (Bio-Rad, USA) in a transfer buffer. Membranes were blocked by incubation in 5% skim-milk (diluted in Tris-buffered saline and 0.2% Tween-20) for 1.5 h, and probed with appropriate antibodies (1:1000) at 4°C overnight, followed by probing with second antibody goat anti-rabbit IgG-HRP and goat anti-mouse IgG-HRP diluted with TBST to 1:3000, then shake membrane at RT for 1.5 h. Detection by enzyme-linked chemiluminescence (ECL) was performed according to the manufacturer's protocol. Results were analyzed quantitatively using Chemiluminescent and Fluorescent Imaging System.

### Immunohistochemistry

IHC analysis was performed using the DAKO LSAB kit (DAKO A/S, Glostrup, Denmark). Briefly, tissue sections were deparaffinized, rehydrated and incubated with 3% H_2_O_2_ in methanol for 15 min at RT. The antigen was retrieved at 95°C for 20 min using a 0.01 M sodium citrate buffer (pH 6.0). The slides were then incubated with the primary antibody at 4°C overnight. After incubation with the secondary antibody at RT for 1 h, immunostaining was developed using DAB, and the slides were counterstained with hematoxylin.

Two pathologists (Lin Z & Jin T) who did not possess knowledge of the clinical data examined and scored all tissue specimens. In case of discrepancies, a final score was established by reassessment by both pathologists on a double-headed microscope. Briefly, the IHC staining for DEK, E-cadherin, Vimentin, VEGF, CD34, and Ki67 were semi-quantitatively scored as ‘–’ (negative) (no or less than 5% positive cells), ‘+’ (5-25% positive cells), ‘++’ (26-50% positive cells) and ‘+++’ (more than 50% positive cells). The cytoplasmic expression pattern was considered as positive staining. Tissue sections scored as ‘++’ and ‘+++’ were considered as strong positives (overexpression) of DEK, E-cadherin, Vimentin, VEGF, CD34, Ki67 protein.

### Capillary tube formation analyses

HUVECs from American Type Culture Collection were cultured in endothelial cell growth medium (Invitrogen). One hundred microliter of undiluted matrigel was layered in a 96-well plate for 4 h. Then HUVEC cells (1×10^5^ cells/mL) were trypsinized, washed with PBS, and added to the precoated 96-well plates together with the indicated CM treatment. Eighteen hours later, the tubes were observed and photographed at OLYMPUS BX53. The Number of vessel branch points of tube per field was counted from the digital images. Results are expressed as means ± S.D.

### Vasculogenic mimicry analysis

96-well plates were coated with 60 μL Matrigel solution diluted as a 1:1 mixture of High Concentration Matrigel (BD Bioscience) and culture medium without FBS, penicillin and streptomycin solution at 4°C. The plate was allowed to polymerize for one hour at 37°C. MDA-MB-231 and MDA-MB-468 cells, transfected with si-DEK 2, si-DEK 3 and si-Control in DMEM medium without FBS, were seeded at density of 1.5×10^4^ per well. After incubation at 37°C for 6 h, three randomly selected fields of each well were photographed. The vasculogenic mimicry index was calculated using the same method as microtubule formation assay described above.

### CAM assay

In brief, fertilized chicken eggs were kept in a humidified incubator at 37°C for 3 days. Approximately 4-5 mL egg albumin was removed with a hypodermic needle. A 2.5-cm diameter window was made with a razor and a 1% solution of methylcellulose containing various tumor cell CM was loaded inside a silicon ring that was placed onto the surface of CAM. Two days later, 2-3 mL intralipose was injected beneath the CAM, and the membrane was observed at 10×under a microscope and the total number of vessel branch points directly beneath the disc was counted.

### *In vivo* tumorigenesis and metastasis assays

BALB/c nude mice (4-6 weeks old) were purchased from the Vital River Laboratory Animal Technology Co. Ltd., (Beijing, China). All mice were housed in specific pathogen-free conditions following the guidelines of the Institutional Animal Care. To assess the effect of DEK on tumorigenicity *in vivo*, MDA-MB-231 cells (1×10^7^ cells) with si-control or si-DEK 2 were subcutaneously injected into the left flank of the mice. Xenograft growth was monitored every week using a caliper. Tumor volume (V) was monitored by measuring the length (L) and width (W) of the tumor with calipers and was calculated with the formula V= (L×W^2^)×0.5. Immunohistochemical staining for DEK, Vimentin, E-cadherin, VEGF, CD34, and Ki67 was performed on sections from the tumor. All experiments were performed in keeping with the procedures and protocols of the Animal Ethics Committee of Yanbian University.

To produce experimental metastases, MDA-MB-231 cells (1×10^6^) with si-control or si-DEK 2 were harvested in serum-free DMEM medium and injected into the tail vein of nude mice (7 mice/group). The mice were sacrificed after 6 weeks and the lungs were surgically excised and stained with H&E. Lung metastatic lesions were evaluated under a dissecting microscope (×200 magnification).

### Statistical analysis

The data analysis was performed using SPSS 17.0 software and GraphPad Prism 6.0 software. Associations between Tiam1 expression and clinicopathological features were evaluated by a Chi-square test and Fisher's exact test. The Kaplan-Meier method was used for analysis of survival curves, and statistical significance was assessed using the Log-rank test. Cox proportional hazards regression model was used to examine univariate and multivariate hazard ratios for the study variables. Group comparisons for continuous data were done by Mann-Whitney U Test or one-way ANOVA. Biochemical experiments were performed in triplicate and a minimum of three independent experiments were evaluated. The value of *P*<0.05 was considered statistically significant.

## SUPPLEMENTARY MATERIALS FIGURES


