# Supplementary material for: DEK promoted EMT and angiogenesis through regulating PI3K/AKT/mTOR pathway in triple-negative breast cancer
Source: Oncotarget. 2017 Oct 17;8(58):98708–22. doi: 10.18632/oncotarget.21864 (PMC5716761; doi:10.18632/oncotarget.21864)
Supplement: Supplementary file 1 [file oncotarget-08-98708-s001.pdf]

DEK promoted EMT and angiogenesis through regulating PI3K/AKT/mTOR pathway in triple-negative breast cancer

SUPPLEMENTARY MATERIALS

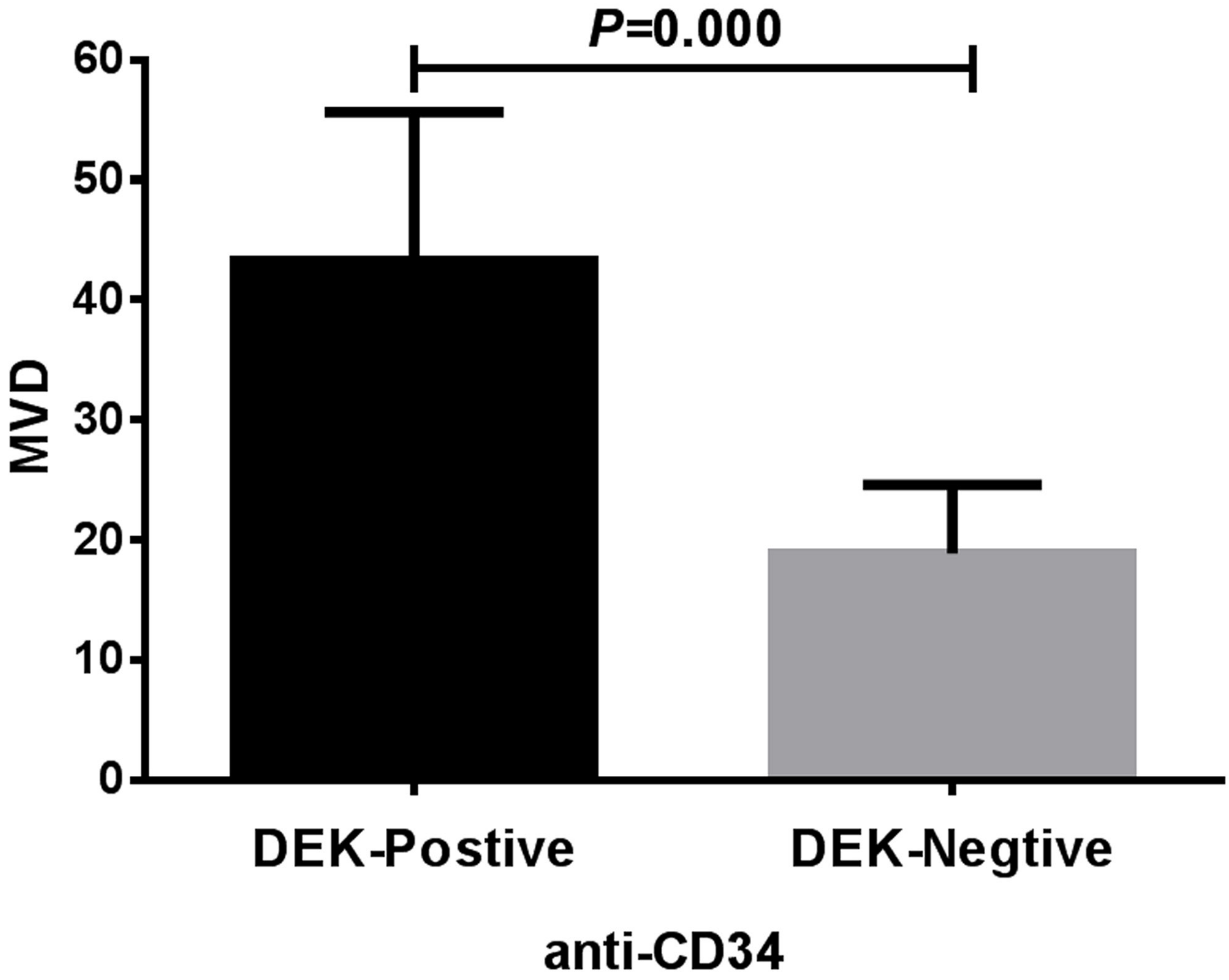

Supplementary Figure 1: The numbers of positively CD34 (MVD) stained cells in TNBC tissues.

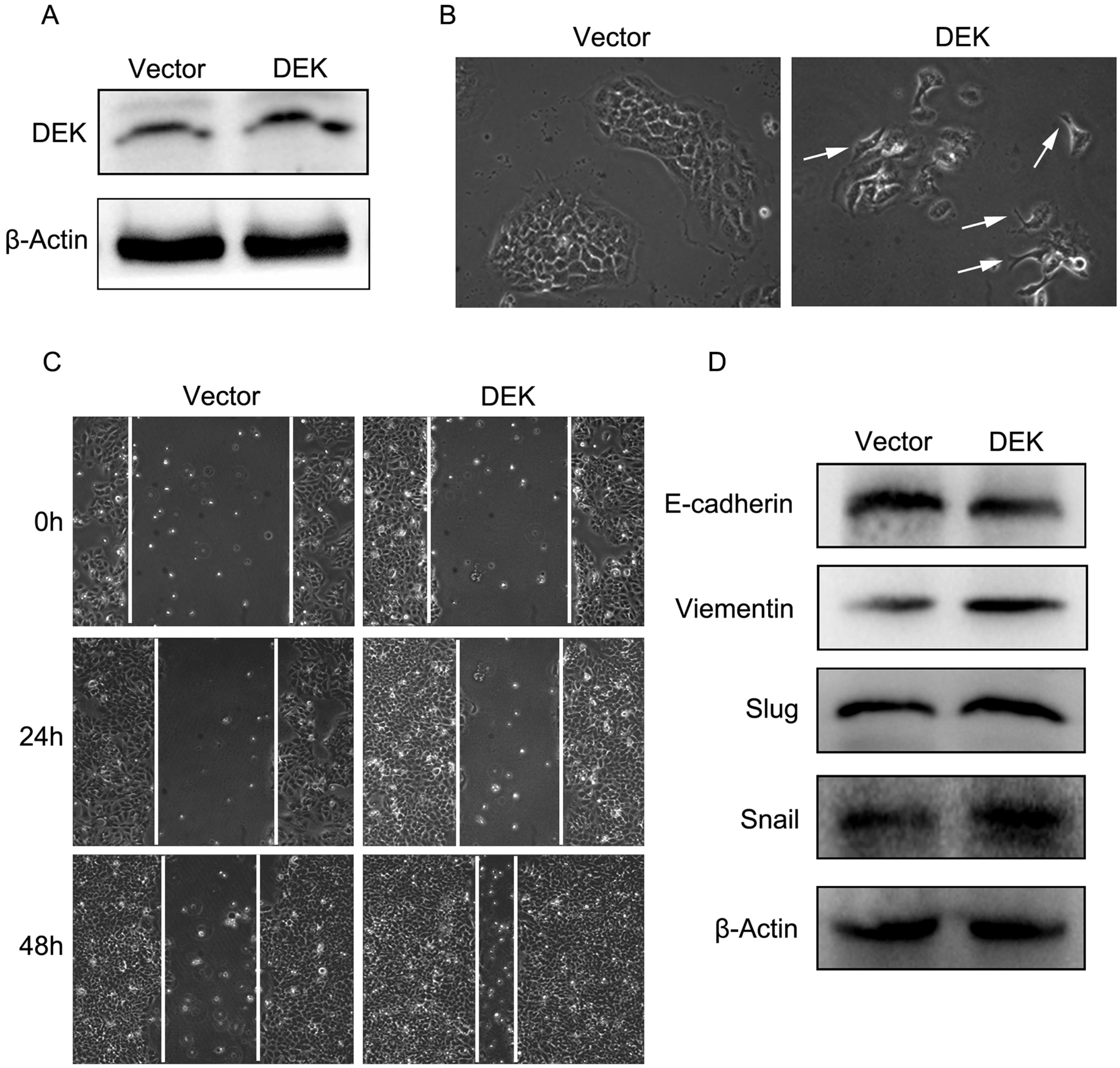

Supplementary Figure 2: DEK upregulation promotes tumorigenic phenotypes in MCF10A cells.

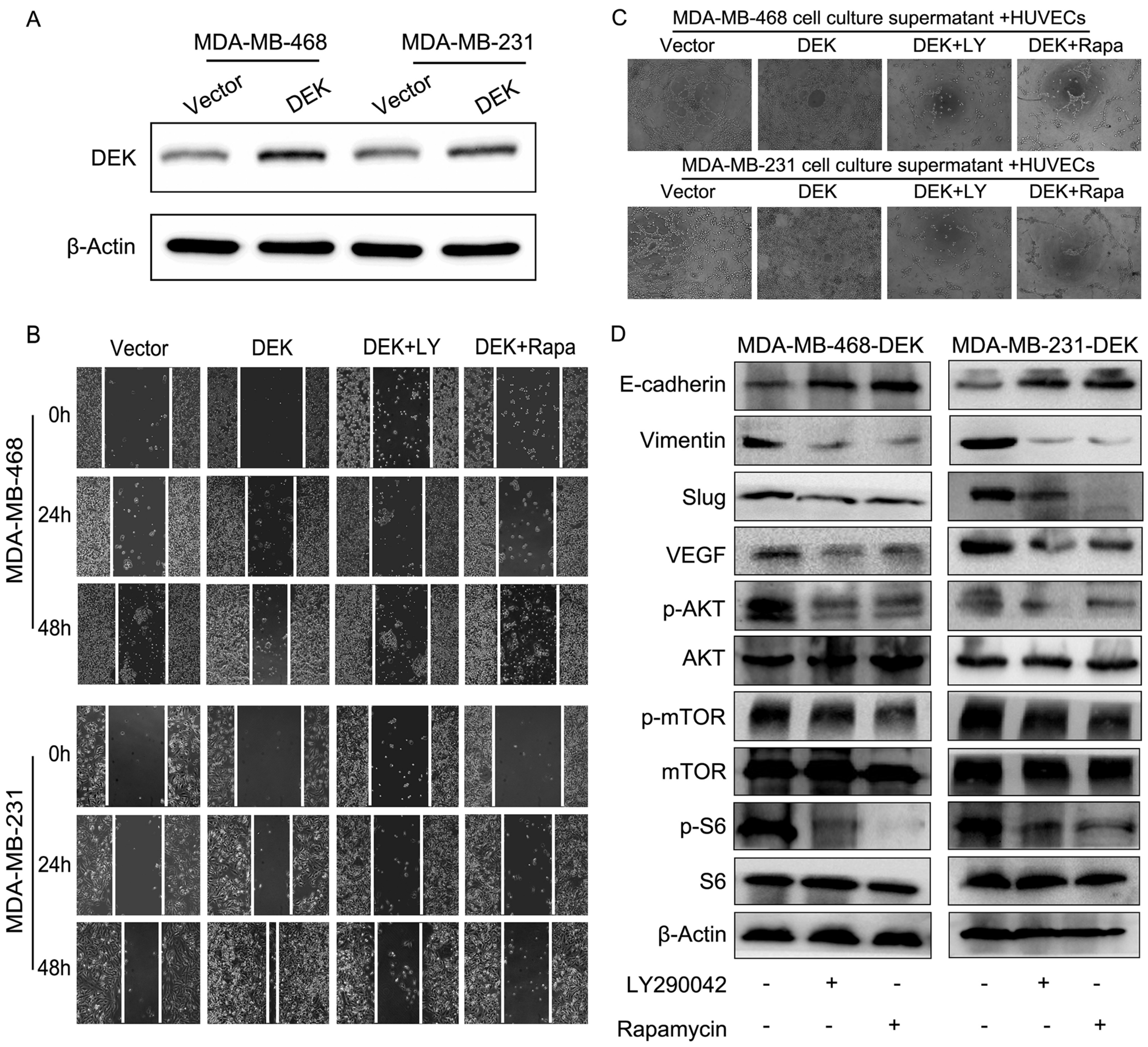

Supplementary Figure 3: DEK upregulation promotes migration, angiogenesis, and EMT through PI3K/AKT/mTOR signaling pathways in TNBC cells.

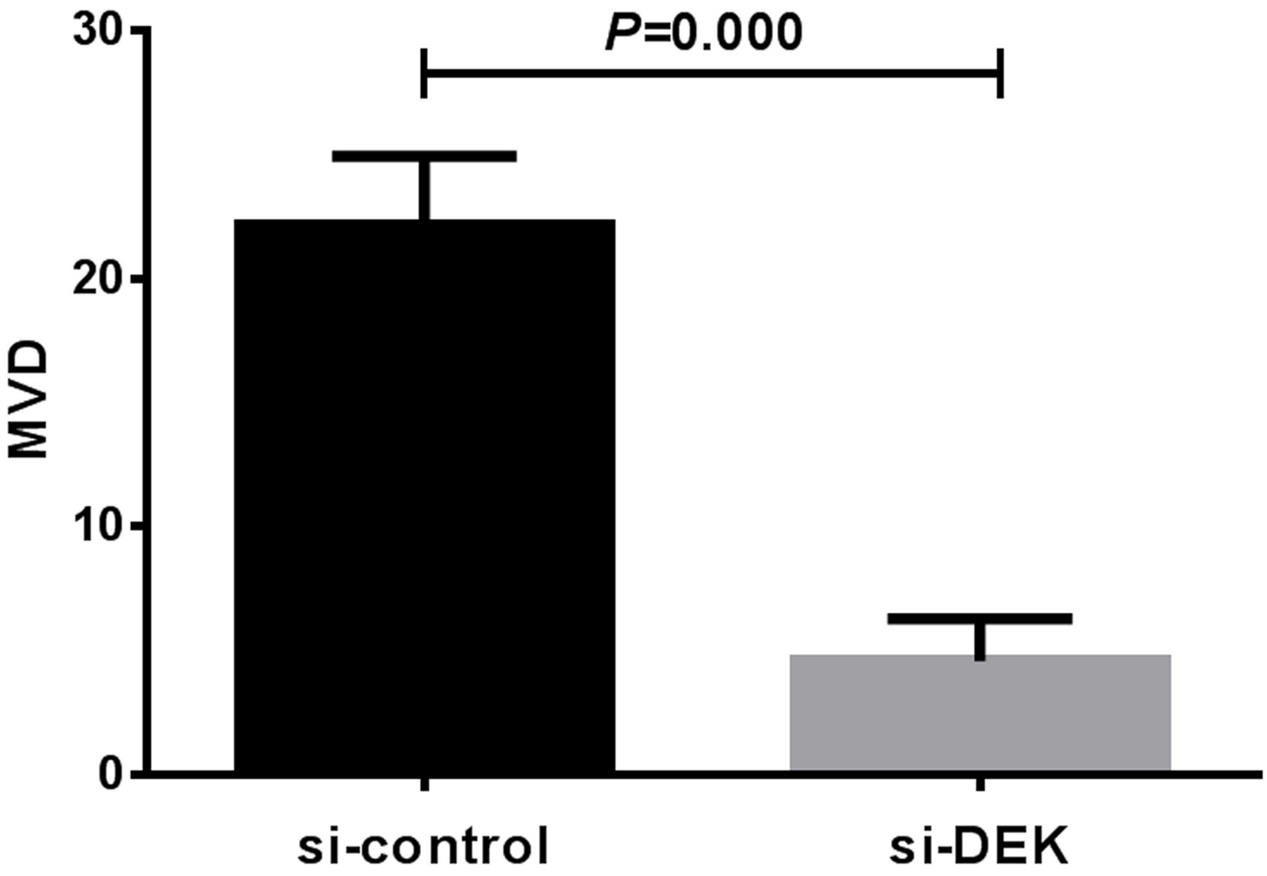

Supplementary Figure 4: The numbers of positively CD34 stained cells in xenograft tumor tissues with si-control and si-DEK.
